# Di-μ-chlorido-bis­{chlorido[(*R*)/(*S*)-1,5-di­phenyl-3-(2-pyridyl-κ*N*)-2-pyrazoline-κ*N* ^2^]zinc(II)}

**DOI:** 10.1107/S1600536810026127

**Published:** 2010-07-07

**Authors:** Miquel Barceló-Oliver, Angel Terrón, Angel García-Raso, Iztok Turel, Marta Morell

**Affiliations:** a Department of Chemistry and IUNICS, Universitat de les Illes Balears, Campus UIB, Cta. Valldemossa km 7.5, Ed. Mateu Orfila i Rotger, E-07122 Palma de Mallorca, Spain; b Faculty of Chemistry and Chemical Technology, University of Ljubljana, Slovenia; c Farchbereich Chemie, Universität Dortmund, 44221 Dortmund, Germany

## Abstract

In the centrosymmetric binuclear title compound, [Zn_2_Cl_4_(C_20_H_17_N_3_)_2_], the coordination geometry of the Zn^II^ ion can be described as a distorted ZnN_2_Cl_3_ trigonal bipyramid (τ = 0.89), arising from the *N*,*N*′-bidentate ligand, a terminal chloride ion and two bridging chloride ions. The N atoms occupy one axial and one equatorial site and the terminal chloride ion occupies an equatorial site. The dihedral angle between the pyridine and pyrazole rings is 12.8 (2)°. In the crystal, aromatic π–π stacking [centroid–centroid separations = 3.812 (3) and 3.848 (3) Å] and C—H⋯Cl and C—H⋯π inter­actions help to establish the packing.

## Related literature

For background to the biochemistry of zinc, see: Casas *et al.* (2002 ▶). For the synthesis of the ligand, see: Barceló-Oliver *et al.* (2010 ▶). For the fluorescent properties of related ligands, see: Bissell *et al.* (1993 ▶); Silva *et al.* (1997 ▶); Wang *et al.* (2001*a* ▶,*b* ▶). For stability constants, see: Yamasaki & Yasuda (1956 ▶). For geometrical analysis, see: Addison *et al.* (1984 ▶). For a description of the Cambridge Structural Database, see: Allen (2002 ▶).
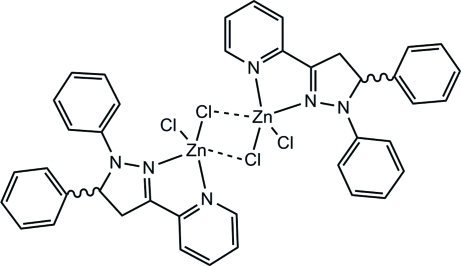


## Experimental

### 

#### Crystal data

[Zn_2_Cl_4_(C_20_H_17_N_3_)_2_]
*M* *_r_* = 871.31
Monoclinic, 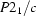

*a* = 14.600 (3) Å
*b* = 8.6200 (17) Å
*c* = 19.524 (7) Å
β = 129.494 (18)°
*V* = 1896.2 (10) Å^3^
*Z* = 2
Mo *K*α radiation
μ = 1.59 mm^−1^
*T* = 150 K
0.21 × 0.15 × 0.04 mm

#### Data collection

Enraf–Nonius KappaCCD diffractometer
Absorption correction: multi-scan (*SADABS*; Bruker, 2000 ▶) *T*_min_ = 0.732, *T*_max_ = 0.939
17229 measured reflections
3467 independent reflections
2206 reflections with *I* > 2σ(*I*)
*R*_int_ = 0.096

#### Refinement

*R*[*F* ^2^ > 2σ(*F* ^2^)] = 0.051
*wR*(*F* ^2^) = 0.095
*S* = 1.04
3467 reflections
235 parameters
H-atom parameters constrained
Δρ_max_ = 0.49 e Å^−3^
Δρ_min_ = −0.41 e Å^−3^

### 

Data collection: *COLLECT* (Nonius, 1998 ▶); cell refinement: *DENZO* and *SCALEPACK* (Otwinowski & Minor, 1997 ▶); data reduction: *DENZO* and *SCALEPACK*; program(s) used to solve structure: *SHELXS97* (Sheldrick, 2008 ▶); program(s) used to refine structure: *SHELXL97* (Sheldrick, 2008 ▶); molecular graphics: *ORTEP-3 for Windows* (Farrugia, 1997 ▶); software used to prepare material for publication: *SHELXL97* and *PLATON* (Spek, 2009 ▶).

## Supplementary Material

Crystal structure: contains datablocks I, global. DOI: 10.1107/S1600536810026127/hb5500sup1.cif

Structure factors: contains datablocks I. DOI: 10.1107/S1600536810026127/hb5500Isup2.hkl

Additional supplementary materials:  crystallographic information; 3D view; checkCIF report

## Figures and Tables

**Table:** 

Zn1—N32	2.048 (3)
Zn1—Cl1	2.2153 (13)
Zn1—Cl2^i^	2.2534 (13)
Zn1—N2	2.340 (3)
Zn1—Cl2	2.7080 (13)

**Table:** 

N32—Zn1—Cl1	111.57 (9)
N32—Zn1—Cl2^i^	126.79 (9)
Cl1—Zn1—Cl2^i^	121.63 (5)
N32—Zn1—N2	76.10 (13)
Cl1—Zn1—N2	105.40 (9)
Cl2^i^—Zn1—N2	89.25 (9)
N32—Zn1—Cl2	90.04 (10)
Cl1—Zn1—Cl2	94.88 (5)
Cl2^i^—Zn1—Cl2	86.03 (5)
N2—Zn1—Cl2	158.51 (9)
Zn1^i^—Cl2—Zn1	93.97 (5)

Symmetry code: (i) 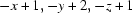.

**Table 2:** Hydrogen-bond geometry (Å, °) *Cg*1 is the centroid of the C51–C56 ring.

*D*—H⋯*A*	*D*—H	H⋯*A*	*D*⋯*A*	*D*—H⋯*A*
C5—H5⋯Cl1^ii^	1.00	2.76	3.474 (5)	128
C12—H12⋯Cl1	0.95	2.69	3.635 (7)	174
C33—H33⋯Cl2	0.95	2.66	3.315 (5)	127
C34—H34⋯*Cg*1^iii^	0.95	2.65	3.557 (7)	159

Symmetry codes: (ii) 
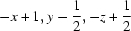
; (iii) 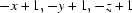.

**Fig. 1.**
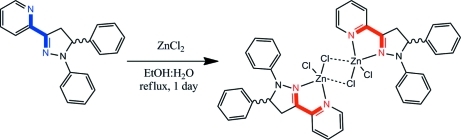
Reaction scheme where the s-trans conformation on the ligand is depicted by a bold blue line and the s-cis conformation found after coordination to the ZnII ion is depicted also with bold red lines.

**Fig. 2.**
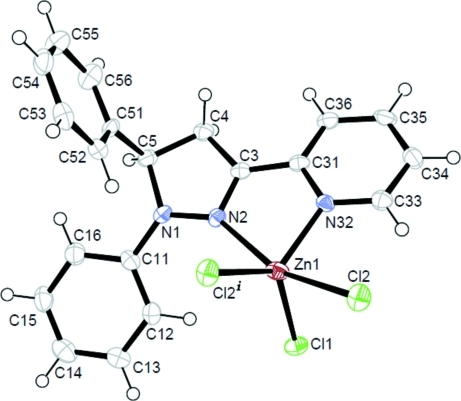
View (50% probability) of the asymmetric unit of (I). The other half of the complex is related by an inversion center, sharing the two Cl2 atoms.

**Fig. 3.**
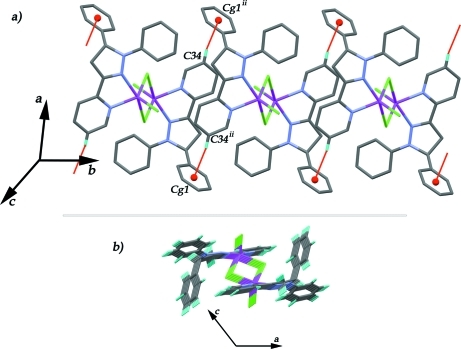
Representation of the C–H···π interaction in (I): a) the interaction forms a one-dimensional chain in the b direction; b) view along the b axis.

## supplementary crystallographic information

### Comment

The interest in Zn^II^ detection is due to the importance of this cation in living systems, because is the second (following iron) most abundant transition metal ion in human body (Casas *et al.*, 2002). The fact that the Zn^II^ is a d^10^ ion, diamagnetic and lacking d-d electronic transitions precludes usual techniques as electronic spectroscopy, e.p.r. or magnetic measurements for studying its compounds. Moreover, the major naturally occurring isotopes have zero nuclear spin and thus, the ion is NMR silent. For these reasons fluorescent probes to determine the "free" or "available" zinc(II) concentrations in living systems should be useful alternatives.

Due to their inner fluorescence properties, 1,3,5-triaryl-^2^Δ-pyrazolines have been utilized as fluorescence probes in some elaborated chemosensors (Bissell *et al.*, 1993 and Silva *et al.*, 1997). On the other hand, the fluorescent 3-(2-pyridyl) analogues of triarylpyrazolines themselves can serve as *N,N*'-type bidentate ligands for metal ions. Wang *et al.* (2001) have reported that pyridylpyrazoline derivatives show specific fluorescent behaviour towards the Zn^2+^ ion among divalent transition metal ions, specially for the 5-(4-cyanophenyl)-1-phenyl-3-(2-pyridyl)-^2^Δ-pyrazoline derivative. Stability constant in acetonitrile for the Zn*L*_2_ complex was determined as 3.4 10^11^ with a clear selectivity to other divalent ions as copper(II) (Wang *et al., *2001). Nevertheless, no references exist in the literature related to structural studies of this type of compounds and metal ions, except for the ternary complex of bis-(2,2'-bipyridyl)ruthenium(II) and a non-fluorescent pyrazoline analogue, the 1-phenyl-3-(2-pyridyl)-5-(4-nitrophenyl)-^2^Δ-pyrazoline (refcode XOCXIW, Wang *et al., *2001) with no coordinates available at Cambridge Structural Database (CSD, Version 5.31, February 2010 update; Allen, 2002). In the present paper, a crystallographic study of the Zn^II^ complex of the fluorescent ligand 1,5-diphenyl-3-(2-pyridyl)-^2^Δ-pyrazoline (Barceló-Oliver *et al.*, 2010) is performed.

A comparison between the previously described ligand (Barceló-Oliver *et al.*, 2010) and its Zn^II^ complex permits to observe a conformational modification related to the pyridine-pyrazoline moiety, where the s-*trans* conformation found in the ligand changes to a s-*cis* disposition in the complex (See Fig. 1). This feature is common in 2,2'-bipyridyl ligands.

Contrarily to previously mentioned 1:2 Zn*L*_2_ complex (Wang *et al., *2001) in our hands, reaction between ZnCl_2_ and 1,5-diphenyl-3-(2-pyridyl)-^2^Δ-pyrazoline only yields a dimeric 1:1 [(LZnCl)_2_Cl_2_] compound. {Zn(Cl)((*R*)-1,5-diphenyl-3-(2-pyridil)-^2^Δ-pyrazoline)}(µ-Cl)_2_{Zn(Cl)((*S*)-1,5-diphenyl-3-(2-pyridil)-^2^Δ-pyrazoline)}] (I) crystallizes as a dimer with an inversion center between the chloride ligands relating the two monomers. We have not isolated any Zn*L*_2_ complex, contrarily to what happen with Zn^II^ and 1,10-phenanthroline or 2,2'-bipyridyne, which have stability constants about 1 10^17 ^and 2 10^13^ respectively (Yamasaki & Yasuda, 1956).

The dimer is formed through two bridged chlorido anions linked with long (Zn1–Cl2) and short (Zn1–Cl2^i^) distances to the Zn^II^ metal ions (see Fig. 2 and Table 1). Moreover, pyridine (Zn1–N32) and imino (Zn1–N2) nitrogen atoms and a monodentate chloride ligand (Zn1–Cl1) are coordinated to both metal ions. The geometry around the metallic ion is described by means a distorted trigonal bipyramid [τ = 0.89 (Addison *et al.*, 1984)]. The bite distance between the two bonding nitrogen atoms is 2.716 (7) Å and the dihedral angle between the pyridyl and the pyrazolyl planes is 12.8°.

Within the dimeric complex unit, some hydrogen bonds are found with the chlorine atoms as acceptors: C5 from the pyrazoline ring interacts with Cl1^i^, C12 from the phenyl ring bonded to N1 is in contact with Cl1 and C33 from the pyridine ring interacts with Cl2 (see Table 2 for more details). More in detail, the second hydrrogen bond (C12–H12···Cl1) forces the twist of the phenyl ring respect to the pyrazoline mean plane (N1–C11 bond): the structure of the ligand (Barceló-Oliver *et al.*, 2010) presents an angle between mean planes of 3.47° while this value is 19.63° in the Zn^II^ complex.

On the other hand, two pyrazoline ligands of adjacent complex units form a centrosymmetric couple along the *b* direction of the crystal by means of two C–H···π interactions between pyridine rings, with C34 as donors, and phenyl rings bounded to C5, corresponding to two different molecules (Table 3 and Fig. 3). This interaction yields a one-dimensional chain throrugh the crystal which is also reinforced with intramolecular π–π interactions (Table 4). To complete the structure of the crystal, two-dimensional planes are formed by means of weaker interactions relating the already described one-dimensional chains among them.

This Zn^II^ complex is soluble in chloroform and presents a distinctive fluorescence in this solvent (λ_exc_ = 384 nm and λ_em_ = 480) related to the previously described ligand [λ_exc_ = 397 nm and λ_em_ = 464 nm (Barceló-Oliver *et al.*, 2010)]. For this reason, this ligand could be useful for Zn^II^ determination in low dielectric constant media. No fluorescence is observed with other metal ions such as Cu(II), Ag(I) and Cd(II), which is in agreement to previous literature data (Wang *et al., *2001), possibly due to a metal quenching.

### Experimental

1,5-diphenyl-3-(2-pyridyl)-^2^Δ-pyrazoline (2 mmol) and ZnCl_2_ (1 mmol) were suspended in 60 ml EtOH/H_2_O (50:50) and refluxed for 24 h. The solution was filtered off and left to crystallize. Orange microcrystals were obtained after 2–3 days [yield: 45%]. The complex was recrystallized from acetonitrile and orange hexagonal rods of (I) were obtained. *Anal. Found*: C, 54.97; H, 3.96; N, 9.60. *Calc. for C_40_H_34_Cl_4_N_6_Zn_2_*: C, 55.14; H, 3.93; N, 9.64. *^1^H NMR (CDCl_3_) (p.p.m.)*: δ 8.70 [bd, 2H, H(33), J = 5.0 Hz], 8.04 [bd, 2H, H(36), J = 7.8 Hz], 7.60 [bt, 2H, H(36), J = 6.3 Hz], 7.38 [m, 20H, H(arom.)], 7.30 [bd, 2H, H(34)], 5.69 [dd, 2H, H(5), J*_cis_* = 8.5, J*_trans_* = 12.8 Hz], 3.99 [dd, 2H, H(4a), J*_trans_* = 12.8, J_gem_ = 17.9 Hz], 3.31 [dd, 2H, H(4 b), J*_cis_* = 8.5, J_gem_ = 17.9 Hz]. *IR (cm^-1^)*: 411w, 452vw, 501w, 539w, 574vw, 652w, 674*m*, 698*m*, 702*m*, 756 s, 763*m*, 782 s, 872*m*, 898*m*, 1002w, 1024w, 1041w, 1082w, 1153vs, 1174 s, 1207w, 121w, 1272*m*, 1321*m*, 1333 s, 1344 s, 1401vs, 1439*m*, 1452*m*, 1491vs, 1507*m*, 1535 s, 1597 s, 1608 s. *UV-vis*.: λ_max_ = 374 nm in EtOH.

### Refinement

All H atoms were placed in geometrically calculated positions and refined using a riding model, with C—H = 0.95–1.00 Å and *U*_iso_(H) = 1.2Ueq(C).

### Crystal data

**Table:**

[Zn_2_Cl_4_(C_20_H_17_N_3_)_2_]	*F*(000) = 888
*M**_r_* = 871.31	*D*_x_ = 1.526 Mg m^−^^3^
Monoclinic, *P*2_1_/*c*	Mo *K*α radiation, λ = 0.71073 Å
Hall symbol: -P 2ybc	Cell parameters from 2226 reflections
*a* = 14.600 (3) Å	θ = 3–25.5°
*b* = 8.6200 (17) Å	µ = 1.59 mm^−^^1^
*c* = 19.524 (7) Å	*T* = 150 K
β = 129.494 (18)°	Hexagon, orange
*V* = 1896.2 (10) Å^3^	0.21 × 0.15 × 0.04 mm
*Z* = 2	

### Data collection

**Table:**

Enraf–Nonius KappaCCD diffractometer	3467 independent reflections
Radiation source: fine-focus sealed tube	2206 reflections with *I* > 2σ(*I*)
graphite	*R*_int_ = 0.096
profile fitted /o scans	θ_max_ = 25.3°, θ_min_ = 3.2°
Absorption correction: multi-scan (*SADABS*; Bruker, 2000)	*h* = −16→17
*T*_min_ = 0.732, *T*_max_ = 0.939	*k* = −9→10
17229 measured reflections	*l* = −23→23

### Refinement

**Table:**

Refinement on *F*^2^	Primary atom site location: structure-invariant direct methods
Least-squares matrix: full	Secondary atom site location: difference Fourier map
*R*[*F*^2^ > 2σ(*F*^2^)] = 0.051	Hydrogen site location: inferred from neighbouring sites
*wR*(*F*^2^) = 0.095	H-atom parameters constrained
*S* = 1.04	*w* = 1/[σ^2^(*F*_o_^2^) + (0.035*P*)^2^ + 0.9808*P*] where *P* = (*F*_o_^2^ + 2*F*_c_^2^)/3
3467 reflections	(Δ/σ)_max_ < 0.001
235 parameters	Δρ_max_ = 0.49 e Å^−^^3^
0 restraints	Δρ_min_ = −0.40 e Å^−^^3^

### Special details

**Table:**

Geometry. All s.u.'s (except the s.u. in the dihedral angle between two l.s. planes) are estimated using the full covariance matrix. The cell s.u.'s are taken into account individually in the estimation of s.u.'s in distances, angles and torsion angles; correlations between s.u.'s in cell parameters are only used when they are defined by crystal symmetry. An approximate (isotropic) treatment of cell s.u.'s is used for estimating s.u.'s involving l.s. planes.
Refinement. Refinement of *F*^2^ against ALL reflections. The weighted *R*-factor *wR* and goodness of fit *S* are based on *F*^2^, conventional *R*-factors *R* are based on *F*, with *F* set to zero for negative *F*^2^. The threshold expression of *F*^2^ > 2σ(*F*^2^) is used only for calculating *R*-factors(gt) *etc*. and is not relevant to the choice of reflections for refinement. *R*-factors based on *F*^2^ are statistically about twice as large as those based on *F*, and *R*- factors based on ALL data will be even larger.

### Fractional atomic coordinates and isotropic or equivalent isotropic displacement parameters (Å^2^)

**Table:**

	*x*	*y*	*z*	*U*_iso_*/*U*_eq_	
Zn1	0.47494 (4)	0.89666 (6)	0.40806 (3)	0.02521 (17)	
Cl1	0.36221 (10)	1.02812 (12)	0.28182 (6)	0.0255 (3)	
Cl2	0.36063 (9)	0.99842 (13)	0.46713 (7)	0.0284 (3)	
N1	0.6991 (3)	0.7462 (4)	0.4103 (2)	0.0244 (9)	
N2	0.5954 (3)	0.7387 (4)	0.3970 (2)	0.0190 (8)	
C3	0.5733 (3)	0.5958 (5)	0.4026 (2)	0.0189 (9)	
C4	0.6604 (3)	0.4843 (5)	0.4129 (3)	0.0229 (10)	
H4A	0.6252	0.428	0.3569	0.027*	
H4B	0.6894	0.4085	0.4609	0.027*	
C5	0.7599 (3)	0.5942 (5)	0.4370 (3)	0.0232 (10)	
H5	0.7833	0.5694	0.3999	0.028*	
C11	0.7316 (4)	0.8767 (5)	0.3875 (2)	0.0232 (10)	
C12	0.6547 (4)	1.0030 (5)	0.3432 (3)	0.0243 (10)	
H12	0.5781	1.0006	0.3271	0.029*	
C13	0.6903 (4)	1.1309 (5)	0.3230 (3)	0.0272 (11)	
H13	0.6384	1.2172	0.294	0.033*	
C14	0.8002 (4)	1.1353 (6)	0.3444 (3)	0.0339 (12)	
H14	0.8239	1.2241	0.3303	0.041*	
C15	0.8750 (4)	1.0108 (6)	0.3860 (3)	0.0346 (12)	
H15	0.9501	1.0133	0.3997	0.041*	
C16	0.8428 (4)	0.8811 (6)	0.4085 (3)	0.0307 (11)	
H16	0.8959	0.796	0.438	0.037*	
C31	0.4686 (4)	0.5567 (5)	0.3919 (2)	0.0205 (10)	
N32	0.4102 (3)	0.6778 (4)	0.3938 (2)	0.0187 (8)	
C33	0.3083 (4)	0.6496 (5)	0.3775 (3)	0.0245 (11)	
H33	0.2671	0.7341	0.3781	0.029*	
C34	0.2600 (4)	0.5029 (5)	0.3597 (3)	0.0282 (11)	
H34	0.1862	0.4874	0.3472	0.034*	
C35	0.3201 (4)	0.3800 (5)	0.3603 (3)	0.0290 (11)	
H35	0.2896	0.2775	0.3496	0.035*	
C36	0.4260 (4)	0.4078 (5)	0.3769 (3)	0.0253 (10)	
H36	0.4692	0.3241	0.3779	0.03*	
C51	0.8688 (3)	0.5881 (5)	0.5352 (3)	0.0220 (10)	
C52	0.8970 (4)	0.7056 (5)	0.5938 (3)	0.0248 (11)	
H52	0.8504	0.7973	0.573	0.03*	
C53	0.9939 (4)	0.6892 (6)	0.6834 (3)	0.0320 (12)	
H53	1.0136	0.7702	0.7237	0.038*	
C54	1.0614 (4)	0.5567 (6)	0.7139 (3)	0.0350 (12)	
H54	1.1269	0.5458	0.7753	0.042*	
C55	1.0346 (4)	0.4391 (6)	0.6558 (3)	0.0402 (13)	
H55	1.0813	0.3474	0.6768	0.048*	
C56	0.9389 (4)	0.4562 (5)	0.5664 (3)	0.0335 (12)	
H56	0.9212	0.3764	0.526	0.04*	

### Atomic displacement parameters (Å^2^)

**Table:**

	*U*^11^	*U*^22^	*U*^33^	*U*^12^	*U*^13^	*U*^23^
Zn1	0.0281 (3)	0.0185 (3)	0.0200 (3)	−0.0029 (3)	0.0111 (2)	0.0000 (2)
Cl1	0.0283 (6)	0.0241 (6)	0.0206 (6)	0.0053 (5)	0.0139 (5)	0.0035 (5)
Cl2	0.0256 (6)	0.0318 (7)	0.0241 (6)	−0.0041 (5)	0.0141 (5)	−0.0095 (5)
N1	0.019 (2)	0.025 (2)	0.027 (2)	0.0054 (17)	0.0136 (19)	0.0062 (16)
N2	0.017 (2)	0.021 (2)	0.0155 (18)	0.0000 (16)	0.0085 (17)	−0.0014 (15)
C3	0.022 (2)	0.018 (2)	0.013 (2)	0.001 (2)	0.0099 (19)	0.0001 (19)
C4	0.024 (3)	0.023 (2)	0.017 (2)	0.000 (2)	0.010 (2)	0.0014 (19)
C5	0.025 (2)	0.023 (2)	0.025 (2)	0.007 (2)	0.018 (2)	0.006 (2)
C11	0.026 (3)	0.025 (3)	0.019 (2)	−0.005 (2)	0.014 (2)	−0.001 (2)
C12	0.023 (2)	0.028 (3)	0.023 (2)	−0.004 (2)	0.015 (2)	−0.007 (2)
C13	0.038 (3)	0.025 (3)	0.024 (2)	−0.002 (2)	0.022 (2)	−0.002 (2)
C14	0.044 (3)	0.035 (3)	0.028 (3)	−0.009 (3)	0.025 (3)	−0.002 (2)
C15	0.025 (3)	0.048 (3)	0.031 (3)	−0.005 (3)	0.018 (2)	0.004 (2)
C16	0.026 (3)	0.038 (3)	0.026 (2)	0.000 (2)	0.016 (2)	0.005 (2)
C31	0.024 (3)	0.019 (2)	0.015 (2)	0.003 (2)	0.011 (2)	0.0019 (17)
N32	0.019 (2)	0.019 (2)	0.0163 (18)	−0.0018 (17)	0.0106 (17)	0.0015 (15)
C33	0.025 (3)	0.026 (3)	0.021 (2)	0.001 (2)	0.014 (2)	0.0025 (19)
C34	0.026 (3)	0.029 (3)	0.028 (3)	−0.006 (2)	0.016 (2)	0.001 (2)
C35	0.033 (3)	0.016 (3)	0.032 (3)	−0.002 (2)	0.018 (2)	0.005 (2)
C36	0.027 (3)	0.018 (2)	0.027 (2)	0.004 (2)	0.015 (2)	0.002 (2)
C51	0.018 (2)	0.026 (2)	0.026 (2)	0.006 (2)	0.016 (2)	0.006 (2)
C52	0.024 (3)	0.024 (3)	0.027 (3)	−0.003 (2)	0.017 (2)	0.001 (2)
C53	0.029 (3)	0.037 (3)	0.031 (3)	−0.012 (3)	0.019 (3)	−0.008 (2)
C54	0.021 (3)	0.048 (3)	0.026 (3)	−0.006 (2)	0.010 (2)	0.003 (2)
C55	0.031 (3)	0.041 (3)	0.043 (3)	0.010 (2)	0.021 (3)	0.009 (3)
C56	0.031 (3)	0.041 (3)	0.025 (3)	0.003 (2)	0.017 (2)	−0.003 (2)

### Geometric parameters (Å, °)

**Table:**

Zn1—N32	2.048 (3)	C15—C16	1.388 (6)
Zn1—Cl1	2.2153 (13)	C15—H15	0.95
Zn1—Cl2^i^	2.2534 (13)	C16—H16	0.95
Zn1—N2	2.340 (3)	C31—N32	1.362 (5)
Zn1—Cl2	2.7080 (13)	C31—C36	1.375 (6)
Cl2—Zn1^i^	2.2534 (13)	N32—C33	1.332 (5)
N1—N2	1.365 (4)	C33—C34	1.381 (6)
N1—C11	1.399 (5)	C33—H33	0.95
N1—C5	1.479 (5)	C34—C35	1.371 (6)
N2—C3	1.295 (5)	C34—H34	0.95
C3—C31	1.445 (6)	C35—C36	1.385 (6)
C3—C4	1.501 (5)	C35—H35	0.95
C4—C5	1.537 (6)	C36—H36	0.95
C4—H4A	0.99	C51—C52	1.381 (5)
C4—H4B	0.99	C51—C56	1.385 (6)
C5—C51	1.526 (5)	C52—C53	1.390 (6)
C5—H5	1	C52—H52	0.95
C11—C12	1.399 (6)	C53—C54	1.373 (6)
C11—C16	1.400 (6)	C53—H53	0.95
C12—C13	1.379 (6)	C54—C55	1.380 (6)
C12—H12	0.95	C54—H54	0.95
C13—C14	1.377 (6)	C55—C56	1.385 (6)
C13—H13	0.95	C55—H55	0.95
C14—C15	1.370 (6)	C56—H56	0.95
C14—H14	0.95		
			
N32—Zn1—Cl1	111.57 (9)	C13—C14—H14	120.2
N32—Zn1—Cl2^i^	126.79 (9)	C14—C15—C16	121.1 (4)
Cl1—Zn1—Cl2^i^	121.63 (5)	C14—C15—H15	119.5
N32—Zn1—N2	76.10 (13)	C16—C15—H15	119.5
Cl1—Zn1—N2	105.40 (9)	C15—C16—C11	119.5 (4)
Cl2^i^—Zn1—N2	89.25 (9)	C15—C16—H16	120.2
N32—Zn1—Cl2	90.04 (10)	C11—C16—H16	120.2
Cl1—Zn1—Cl2	94.88 (5)	N32—C31—C36	121.0 (4)
Cl2^i^—Zn1—Cl2	86.03 (5)	N32—C31—C3	116.1 (4)
N2—Zn1—Cl2	158.51 (9)	C36—C31—C3	122.8 (4)
Zn1^i^—Cl2—Zn1	93.97 (5)	C33—N32—C31	118.5 (4)
N2—N1—C11	122.5 (3)	C33—N32—Zn1	123.3 (3)
N2—N1—C5	111.7 (3)	C31—N32—Zn1	117.9 (3)
C11—N1—C5	125.1 (3)	N32—C33—C34	122.8 (4)
C3—N2—N1	109.5 (3)	N32—C33—H33	118.6
C3—N2—Zn1	107.7 (3)	C34—C33—H33	118.6
N1—N2—Zn1	139.7 (3)	C35—C34—C33	119.0 (4)
N2—C3—C31	120.4 (4)	C35—C34—H34	120.5
N2—C3—C4	112.7 (4)	C33—C34—H34	120.5
C31—C3—C4	126.7 (4)	C34—C35—C36	118.8 (4)
C3—C4—C5	101.7 (3)	C34—C35—H35	120.6
C3—C4—H4A	111.4	C36—C35—H35	120.6
C5—C4—H4A	111.4	C31—C36—C35	119.9 (4)
C3—C4—H4B	111.4	C31—C36—H36	120.1
C5—C4—H4B	111.4	C35—C36—H36	120.1
H4A—C4—H4B	109.3	C52—C51—C56	119.3 (4)
N1—C5—C51	112.7 (3)	C52—C51—C5	122.6 (4)
N1—C5—C4	101.5 (3)	C56—C51—C5	118.0 (4)
C51—C5—C4	113.0 (3)	C51—C52—C53	119.8 (4)
N1—C5—H5	109.8	C51—C52—H52	120.1
C51—C5—H5	109.8	C53—C52—H52	120.1
C4—C5—H5	109.8	C54—C53—C52	120.4 (4)
N1—C11—C12	121.4 (4)	C54—C53—H53	119.8
N1—C11—C16	119.6 (4)	C52—C53—H53	119.8
C12—C11—C16	119.0 (4)	C53—C54—C55	120.3 (4)
C13—C12—C11	120.0 (4)	C53—C54—H54	119.9
C13—C12—H12	120	C55—C54—H54	119.9
C11—C12—H12	120	C54—C55—C56	119.3 (5)
C14—C13—C12	120.9 (4)	C54—C55—H55	120.3
C14—C13—H13	119.6	C56—C55—H55	120.3
C12—C13—H13	119.6	C55—C56—C51	120.9 (4)
C15—C14—C13	119.6 (4)	C55—C56—H56	119.6
C15—C14—H14	120.2	C51—C56—H56	119.6
			
N32—Zn1—Cl2—Zn1^i^	126.89 (9)	N1—C11—C16—C15	−179.9 (4)
Cl1—Zn1—Cl2—Zn1^i^	−121.45 (5)	C12—C11—C16—C15	0.7 (6)
Cl2^i^—Zn1—Cl2—Zn1^i^	0	N2—C3—C31—N32	−12.6 (5)
N2—Zn1—Cl2—Zn1^i^	77.7 (2)	C4—C3—C31—N32	172.9 (3)
C11—N1—N2—C3	164.2 (3)	N2—C3—C31—C36	164.7 (4)
C5—N1—N2—C3	−6.7 (4)	C4—C3—C31—C36	−9.8 (6)
C11—N1—N2—Zn1	−39.4 (5)	C36—C31—N32—C33	−2.4 (5)
C5—N1—N2—Zn1	149.7 (3)	C3—C31—N32—C33	175.0 (3)
N32—Zn1—N2—C3	−10.4 (2)	C36—C31—N32—Zn1	−175.8 (3)
Cl1—Zn1—N2—C3	−119.4 (2)	C3—C31—N32—Zn1	1.5 (4)
Cl2^i^—Zn1—N2—C3	117.9 (2)	Cl1—Zn1—N32—C33	−67.2 (3)
Cl2—Zn1—N2—C3	40.8 (4)	Cl2^i^—Zn1—N32—C33	113.1 (3)
N32—Zn1—N2—N1	−167.1 (4)	N2—Zn1—N32—C33	−168.5 (3)
Cl1—Zn1—N2—N1	83.9 (4)	Cl2—Zn1—N32—C33	28.0 (3)
Cl2^i^—Zn1—N2—N1	−38.8 (4)	Cl1—Zn1—N32—C31	105.9 (3)
Cl2—Zn1—N2—N1	−115.9 (4)	Cl2^i^—Zn1—N32—C31	−73.7 (3)
N1—N2—C3—C31	179.4 (3)	N2—Zn1—N32—C31	4.6 (3)
Zn1—N2—C3—C31	15.1 (4)	Cl2—Zn1—N32—C31	−158.8 (3)
N1—N2—C3—C4	−5.4 (4)	C31—N32—C33—C34	0.6 (6)
Zn1—N2—C3—C4	−169.7 (2)	Zn1—N32—C33—C34	173.7 (3)
N2—C3—C4—C5	14.3 (4)	N32—C33—C34—C35	1.3 (6)
C31—C3—C4—C5	−170.8 (3)	C33—C34—C35—C36	−1.4 (6)
N2—N1—C5—C51	−106.1 (4)	N32—C31—C36—C35	2.2 (6)
C11—N1—C5—C51	83.4 (5)	C3—C31—C36—C35	−174.9 (4)
N2—N1—C5—C4	15.0 (4)	C34—C35—C36—C31	−0.3 (6)
C11—N1—C5—C4	−155.6 (3)	N1—C5—C51—C52	7.7 (5)
C3—C4—C5—N1	−16.2 (4)	C4—C5—C51—C52	−106.5 (5)
C3—C4—C5—C51	104.7 (4)	N1—C5—C51—C56	−174.7 (4)
N2—N1—C11—C12	−5.1 (6)	C4—C5—C51—C56	71.1 (5)
C5—N1—C11—C12	164.5 (4)	C56—C51—C52—C53	−1.0 (6)
N2—N1—C11—C16	175.6 (3)	C5—C51—C52—C53	176.5 (4)
C5—N1—C11—C16	−14.9 (6)	C51—C52—C53—C54	−0.3 (6)
N1—C11—C12—C13	178.9 (3)	C52—C53—C54—C55	0.9 (7)
C16—C11—C12—C13	−1.7 (6)	C53—C54—C55—C56	−0.1 (7)
C11—C12—C13—C14	1.3 (6)	C54—C55—C56—C51	−1.2 (7)
C12—C13—C14—C15	0.2 (6)	C52—C51—C56—C55	1.8 (7)
C13—C14—C15—C16	−1.2 (7)	C5—C51—C56—C55	−175.9 (4)
C14—C15—C16—C11	0.7 (7)		

Symmetry codes: (i) −*x*+1, −*y*+2, −*z*+1.

### Hydrogen-bond geometry (Å, °)

**Table:**

Cg1 is the centroid of the C51–C56 ring.

**Table:**

*D*—H···*A*	*D*—H	H···*A*	*D*···*A*	*D*—H···*A*
C5—H5···Cl1^ii^	1.00	2.76	3.474 (5)	128
C12—H12···Cl1	0.95	2.69	3.635 (7)	174
C33—H33···Cl2	0.95	2.66	3.315 (5)	127
C34—H34···Cg1^iii^	0.95	2.65	3.557 (7)	159

Symmetry codes: (ii) −*x*+1, *y*−1/2, −*z*+1/2; (iii) −*x*+1, −*y*+1, −*z*+1.

### Table 3 Centroid–centroid interactions (Å, °)

Cg2 and Cg3 are the centroids of the C311/N32/C33–C36 and C11–C16 rings, respectively. As defined in PLATON (Spek, 2009), α is the dihedral angle between planes I and J, β is the angle between the CgI->CgJ vector and the normal to the plane I, γ is the angle between the CgI->CgJ vector and the normal to the plane J, CgI-Perp is the perpendicular distance of CgI from ring J, CgJ-Perp is the perpendicular distance of CgJ from ring I, and the slippage S is the distance between CgI and the perpendicular projection of CgJ on ring I.

**Table:**

CgI···CgJ	Cg···Cg	α	β	γ	CgI-Perp	CgJ-Perp	S
Cg2···Cg2^ii^	3.848 (3)	0	22.0	22.0	3.5675 (17)	3.5676 (17)	1.442
Cg2···Cg3^i^	3.812 (3)	17.1 (2)	13.1	27.2	3.3884 (17)	3.7126 (18)	

Symmetry codes: (i) 1-x, -1/2+y, 1/2-z; (ii) 1-x, 1-y, 1-z
